# Stat3 and Gap Junctions in Normal and Lung Cancer Cells

**DOI:** 10.3390/cancers6020646

**Published:** 2014-03-25

**Authors:** Stephanie Guy, Mulu Geletu, Rozanne Arulanandam, Leda Raptis

**Affiliations:** 1 Department of Pathology, Queen’s University, Kingston, ON K7L 3N6, Canada; E-Mail: 11sg27@queensu.ca; 2 Department of Biomedical and Molecular Sciences, Queen’s University, Kingston, ON K7L 3N6, Canada

**Keywords:** signal transducer and activator of transcription-3, gap junctions, connexin43

## Abstract

Gap junctions are channels linking the interiors of neighboring cells. A reduction in gap junctional intercellular communication (GJIC) correlates with high cell proliferation, while oncogene products such as *Src* suppress GJIC, through the Ras/Raf/Erk and other effector pathways. High *Src* activity was found to correlate with high levels of the *Src* effector, Signal Transducer and Activator of Transcription-3 (Stat3) in its tyrosine-705 phosphorylated, *i.e.*, transcriptionally activated form, in the majority of Non-Small Cell Lung Cancer lines examined. However, Stat3 inhibition did not restore GJIC in lines with high *Src* activity. In the contrary, Stat3 inhibition in normal cells or in lines with low *Src* activity and high GJIC eliminated gap junctional communication. Therefore, despite the fact that Stat3 is growth promoting and in an activated form acts like an oncogene, it is actually required for junctional permeability.

## 1. Introduction

Gap junctions are channels linking the interiors of neighboring cells that allow the passage of ions and small metabolites. They consist of integral membrane proteins, the connexin gene family, comprising 21 known members in humans [1]. Connexins play a critical role in the regulation of development, contraction, homeostasis, as well as cell growth and differentiation [2]. 

Connexins are composed of four transmembrane domains, two extracellular loops with six conserved cysteine residues, a cytoplasmic loop and cytoplasmic amino- and carboxy-termini. The carboxy-terminus varies in length and provides sites for protein interactions [2]. Connexin-43 (Cx43) is a ubiquitous connexin which is present in at least 43 tissues and 46 different cell types, and is the predominant connexin in most cultured cell lines [3,4]. Cx43 has an unusually short half-life (1–3 h), both in cell culture and in tissues, with a high turnover [5]. Like most integral membrane proteins, Cx43 is cotranslationally inserted into the endoplasmic reticulum during its biosynthesis, where interloop disulphide bonds form. Cx43 then enters the trans-Golgi network, where it oligomerises into hexamers, called connexons, which migrate to the cell surface where they coalesce at sites nucleated for gap junction assembly [6,7]. Following their formation, gap junctions are internalised by a mechanism where the entire gap junction or fragments thereoff form a double-membrane structure, termed connexosome, which merges with lysosomes where they are degraded [2,3,6]. 

A number of oncogene products such as v-*Src* [8], the middle tumor antigen of polyoma virus (mT, which transforms through c*Src* activation [9,10]), the activated chaperone Hsp90N [11], activated Ras [12], v-mos [13] and others were shown to interrupt gap junctional, intercellular communication (GJIC). Some of these oncogenes have kinase activity and phosphorylate Cx43 on tyrosine or serine/threonine sites. In fact, Cx43 is highly phosphorylated. Activation of kinases such as protein kinase A (PKA) and protein kinase CK1 increases Cx43 phosphorylation and gap junction transfer, while activation of protein kinase C, p34cdc2 (cyclin B kinase), Erk1/2 and *Src* decrease GJIC [14,15,16]. 

*src* is the first oncogene to be discovered, as the tumor gene of the Rous sarcoma virus. *src* encodes a protein with tyrosine kinase activity (*Src*), which correlates with tyr-418 phosphorylation (*Src*418). *Src* is often activated in cancer. As such, *Src* is one of the best studied targets for cancer therapy. Results from a number of labs indicated that the *Src* kinase can affect Cx43 phosphorylation either directly on tyrosine, or through its effector pathways on serine or threonine. At first the *Src*-homology-3 (SH3) domain of *Src* binds a proline-rich sequence of Cx43, Pro-274 to Pro-280. The proximity between the two proteins facilitates phosphorylation of Cx43 at tyr-265 by *Src*. This creates a binding site for the *Src*, Src-homology-2 (SH2) domain that facilitates phosphorylation of tyr-247 of Cx43, triggering channel closure [8,16]. Still, the role of *Src*, as opposed to its effectors, upon GJIC in cancer is unclear. The *Src* effectors, PLCγ (which activates protein kinase C, PKC) and Erk1/2 also suppress GJIC in their own right, through phosphorylation at serines 368 and 372, or 255, 279 and 282, respectively. In fact, the Ras/Raf/Erk pathway was shown to transduce *Src* signals leading to GJIC suppression [17].

A prominent *Src* effector is the Signal Transducer and Activator of Transcription-3 (Stat3) [18], a key cytoplasmic signal transducer which is often overactive in cancer. A constitutively active form of Stat3, Stat3C, can transform cultured mouse fibroblasts, pointing to an etiological role of Stat3 in cancer [19]. Besides the *Src* family, a number of growth factor receptors such as the EGF family including Her2/ErbB2 and receptors for cytokines such as the IL6 family phosphorylate Stat3 at tyr-705 (Stat3-705). This triggers Stat3 dimerisation through a reciprocal interaction between the SH2 domain and ptyr-705 of Stat3, migration to the nucleus and activation of transcription of specific genes involved in cell division and survival, such as myc, Bcl-xL, Mcl-1, survivin, HGF [20] and others (reviewed in [21]). Recent results from our lab and others demonstrated that engagement of cadherins, cell to cell adhesion molecules, as occurs in cultured cells grown to high densities, can dramatically increase Stat3, tyr-705 phosphorylation and transcriptional activity [22,23,24]. This was found to be triggered by a dramatic increase in the levels of the Rac1 and Cdc42 GTPases through inhibition of their proteasomal degradation, and this leads to transcriptional upregulation of the interleukin-6 (IL6) gene, triggering Stat3, tyr-705 phosphorylation and activation ([22], reviewed in [25,26]). 

Despite the extensive literature on the effect of oncogenes upon GJIC, the effect of Stat3 upon GJIC in cancer cells is unclear. Here, we summarise our recent findings on the effect of the *Src*/Stat3 axis upon GJIC in cells transformed in culture and in lines established from Non-Small Cell Lung Cancer (NSCLC) tumors. Although in the majority of lines high *Src*418 correlated with high Stat3-705 and the absence of GJIC, Stat3 inhibition did not restore communication in any of the lines examined. This is in sharp contrast to inhibition of the Ras pathway, which did increase GJIC [17]. In the contrary, Stat3 inhibition in lines with extensive GJIC eliminated junctional permeability, indicating that, despite the fact that in an activated form it can act as an oncogene [19], Stat3 is actually required for gap junctional communication. 

## 2. GJIC Examination

A reliable method to measure gap junctional permeability is a prerequisite for the investigation of the effect of Stat3 upon GJIC. GJIC examination is usually conducted through the introduction of a fluorescent tracking dye such as Lucifer yellow (LY) by microinjection, scrape-loading [27] or preloading with dye [28], followed by observation of its migration into neighboring cells, or by measuring the recovery of fluorescence after photobleaching [29]. These methods are generally expensive and time-consuming or introduce the potential complication of cellular damage. To overcome these problems, we developed a powerful technique where cells are grown on a glass slide, half of which is coated with electrically conductive, optically transparent, Indium-Tin oxide (ITO) [30]. A LY solution is added to the cells and an electrical pulse delivered. The pulse opens transient pores on the plasma membrane through which LY enters the cell, then rapidly reclose with no detectable damage to the cell. As a result, cells growing on the conductive side of the slide are loaded with LY through electroporation, while cells on the adjoining, non-conductive area do not receive any current, therefore are not permeabilized. The LY can then diffuse through gap junctions into these cells, forming a gradient of fluorescence. Following washing, the cells are observed under phase contrast and fluorescence illumination. Tracer movement can be evaluated several minutes after the electrical pulse, by overlapping the phase contrast and fluorescence images of the cells [9,31,32]. Gap junctional communication can be precisely quantitated in this way, simultaneously and in a large number of cells, without any detectable disturbance to cellular metabolism, presumably because the pores reclose rapidly, so that the cellular interior is restored to its original state. The average number of cells into which LY has transferred, per cell loaded with LY by electroporation is the GJIC value. Normally, the transfer from at least 200 cells is calculated [33,34,35]. The equipment (ACE-100, InSitu Porator apparatus) is available from Cell Projects Ltd. (Harrietsham, Kent, UK).

Electroporation *in situ* was employed to examine the role of Stat3 upon GJIC, both in immortalised rat liver epithelial T51B [36] or mouse lung epithelial type II E10 [37] cells both of which normally have extensive GJIC, and in lung cancer lines or primary tumor cells expressing different levels of activated *Src* [33,38]. 

## 3. Stat3 and GJIC in Cultured, Normal and *Src*-Transformed Cells

### 3.1. Cell Density Upregulates GJIC and Connexin-43 Protein Levels

The formation of gap junctions depends upon cell to cell contact, and the engagement of cadherins into adherens junctions [39]. Therefore, it is not surprising that in immortalised rat liver epithelial T51B or mouse lung E10 cells [37], GJIC showed a substantial increase from approximately 1.1 to 6, when cells were grown to densities from 90% to 3 days post-confluence [33]. Interestingly, the levels of total Cx43 protein also increased dramatically with density and plateaued at ~2 days after 100% confluence. These data indicate that cell to cell contact can trigger a significant increase in GJIC and Cx43 levels. Therefore, in all subsequent experiments GJIC was examined at 3 days post-confluence [33].

### 3.2. Stat3 Does Not Transduce Src Signals to GJIC Suppression

We at first examined the effect of Stat3 upon GJIC in *in vitro* transformed, T51B cells where *Src* was activated through mT expression (T51B-*Src* cells) [36]. Stat3 was downregulated through treatment with the pharmacological inhibitors CPA7 [40,41] or 23I-201 [38,42], or through expression of shRNA with a retroviral vector [36]. Given that Stat3 is an effector of *Src*, a tyrosine kinase with the ability for GJIC suppression, and an oncogene in its own right [19], it was expected that Stat3 might transduce *Src* signals leading to GJIC suppression. However, the results demonstrated that Stat3 downregulation does not restore GJIC, indicating that the high Stat3 activity in T51B-*Src* cells cannot be responsible for their lack of junctional communication. This is in sharp contrast to Ras, whose inhibition restored GJIC in *Src*-transformed rodent fibroblasts, and consistent with findings by Ito *et al.* [17]. 

### 3.3. Stat3 Is a Positive Regulator of GJIC and Cx43 Levels

We next investigated whether Stat3 might, in fact, play a positive role upon GJIC, by examining the effect of Stat3 inhibition in the parental T51B cells. Interestingly, Stat3 downregulation essentially eliminated GJIC in T51B cells [36]. Conversely, expression of the constitutively active form of Stat3, Stat3C [19] increased the already extensive gap junctional communication in T51B cells ([33,43]). Taken together, these data indicate that Stat3 does in fact play a positive role in the maintenance of gap junction function. This is in sharp contrast to Ras, which was shown to suppress GJIC in non-transformed rodent fibroblasts [12]. Therefore, rather than increasing GJIC, Stat3 inhibition eliminates junctional permeability, indicating that Stat3 activity is actually required for gap junction function.

As noted above, the half-life of Cx43 is 1–3 h, while cell density (which increases Stat3-705) causes a dramatic increase in Cx43 levels in non-transformed cells. Interestingly, Stat3 downregulation through CPA7 treatment or shRNA expression caused a dramatic reduction in Cx43 levels in rat liver epithelial T51B, mouse lung E10 cells or rat F111 fibroblasts at all densities examined, concomitant with GJIC reduction. These findings indicate that Stat3 activity is required for the maintenance of Cx43 protein levels [33].

## 4. *Src* as a Stat3 Activator in Non-Small Cell Lung Cancer

### 4.1. Cell Density Increases Stat3, in a *Src*-Independent Manner

Examination of the levels of tyr-418 phosphorylated, *i.e.*, activated *Src* (*Src*418) in a number of NSCLC biopsies revealed the presence of higher *Src* activity than the surrounding, non-tumor lung tissue [44,45]. However, *Src*’s contribution to Stat3 activity in NSCLC lines and primary cells which may express other oncogenes in addition to *Src* is a matter of controversy. Examination of Stat3 levels in certain NSCLC lines demonstrated that *Src* is a major Stat3 activator [46], while in another report [47] *Src* inhibition in different NSCLC lines was found to actually increase Stat3-ptyr705. Since cell density causes a dramatic increase in Stat3-705 [25], differences in confluence might account at least in part for these apparent discrepancies. 

**Figure 1:**
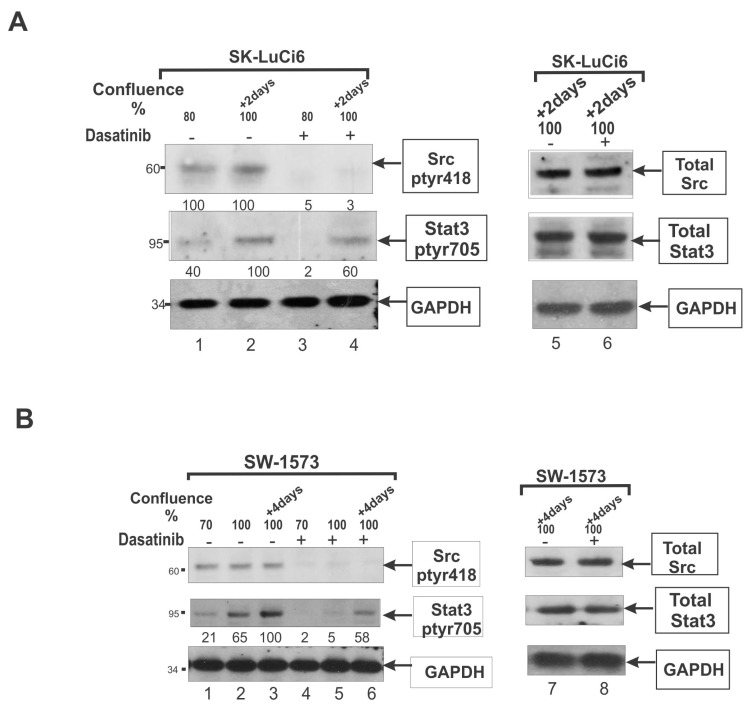
Density-induced, Stat3 activation is independent of *Src*. (**A**) SK-Luci6 cells were grown to densities of 80% confluence or 2 days post-confluence as indicated and treated with Dasatinib (lanes 3,4) or not (lanes 1,2). Detergent cell extracts were probed for *Src*418, Stat3-705, or GAPDH as a loading control. Right panel, lysates were probed for total Src, total Stat3 or GAPDH. Numbers under the lanes refer to densitometry values. Numbers at the left refer to Molecular weight markers; (**B**) SW-1573 cells were grown to the indicated densities and treated with Dasatinib (lanes 4–6) or not (lanes 1–3). Detergent cell extracts were probed for *Src*418, Stat3-705, or GAPDH as a loading control. Right panel, lysates were probed for total Src, total Stat3 or GAPDH. Numbers under the lanes refer to densitometry values. Numbers at the left refer to Molecular weight markers.

Despite the fact that *Src* is a potent Stat3 activator, *Src*418 levels did not increase with density in T51B or E10 cells, or in the NSCLC lines SK-LuCi6 (Figure 1A) and QUDB that have low *Src* levels, before or after expression of activated *Src* (lines T51B-*Src*, E10-*Src*, SK-LuCi6-*Src* and QUDB-*Src*, respectively). Moreover, *Src* inhibition with the inhibitors dasatinib (Figure 1A,B) or PD180970, or downregulation through expression of a *Src*DN mutant with an adenovirus vector [24,38], or genetic ablation of *Src* as well as the related Yes and Fyn genes [23] did not reduce the density-dependent, Stat3 upregulation. Taken together, these findings indicate that the density-mediated, Stat3 activation is independent of *Src*. Therefore, to avoid the confounding factor of density upon Stat3-705, the correlation between *Src*418 and Stat3-705 was examined at 50% confluence, in a number of NSCLC lines.

### 4.2. Src Is a Major Stat3 Activator in Certain NSCLC Lines

Besides *Src*, Stat3 is known to be a prominent effector of other cytokine and membrane tyrosine kinase receptors, which may be present in an activated form in NSCLC cells and upregulate Stat3 activity. Therefore, to examine the effect of *Src* specifically upon Stat3 the correlation between *Src*418 and Stat3-705 levels was at first assessed in a number of NSCLC lines. Nine lines displayed high *Src*418 levels (100% to 25% of SK-LuCi6-*Src*), which were found to correlate with their levels of Stat3-705 (Table 1) [33,38] pointing to the possibility that *Src* may be a significant contributor to Stat3 activity in these lines. At the same time, two lines with low *Src*418 levels (QUDB and SK-LuCi6) had low Stat3-705. In sharp contrast however, line LC-T displayed high Stat3-705 despite the fact that *Src*418 was undetectable. Similarly, SK-MES cells had intermediate *Src*418 levels (25% of SK-LuCi6-*Src*), although Stat3-705 was at 90%, and FR-E cells with *Src*418 of 25% displayed Stat3-705 of 70%, indicating that other, *Src*-independent factor(s) must also be responsible for the high Stat3-ptyr705 in these lines (Table 1).

Examination of the actual contribution of *Src* to Stat3-705 levels was performed by assessing the ability of the *Src* inhibitors dasatinib or PD180970 to reduce Stat3-705 levels. The results revealed that in sparsely growing cells (e.g., SW-1573) dasatinib caused a dramatic reduction in Stat3-705 (Figure 1B, lane 1 *vs.* 4 and 2 *vs.* 5), pointing to *Src* as a significant Stat3 activator. However, in confluent cultures the reduction was only ~50% (lane 3 *vs.* 6), which further confirms that the density-induced, Stat3 activation is independent of *Src*. Similar results were obtained with 11 lines with high *Src*418 (Table 1). Consistent with this observation, in cells with low *Src*418 (e.g., SK-LuCi6 (Figure 1A), QUDB, SHP-77 and BHE), the Stat3-705 increase observed at high confluence was resistant to dasatinib treatment.

**Table 1:** *Src*, Stat3 and GJIC in lung cancer lines.

Cell line	*Src*^α^ (%)	Stat3 ^α^ (%)		GJIC ^β^
		50% confluent	100 + 3 days	
Lines with high *Src*-418 levels				
SK-LuCi6-*Src*	100 ± 12	100 ± 10	420 ± 33	0.2 ± 0.1
A549	95 ± 11	93 ± 12	320 ± 32	0.3 ± 0.1
SK-Lu1	85 ± 5	90 ± 11	311 ± 23	1 ± 0.2
Calu-1	96 ± 9	100 ± 10	290 ± 12	0.1 ± 0.1
SW-900	100 ± 13	100 ± 12	405 ± 21	0.1 ± 0.1
Calu-6	95 ± 10	90 ± 12	300 ± 18	0.1 ± 0.1
SW-1573	70 ± 9	70 ± 8	180 ± 12	0.2 ± 0.1
WT-E	60 ± 9	30 ± 4	80 ± 11	0.2 ± 0.1
BEN	42 ± 5	60 ± 8	182 ± 25	0.2 ± 0.1
H1299	30 ± 4	30 ± 5	60 ± 18	0.2 ± 0.1
FRE	25 ± 3	70 ± 4	180 ± 25	0.5 ± 0.1
SK-MES	25 ± 2	90 ± 6	248 ± 22	1 ± 0.3
Lines with low *Src*-418 levels				
LCT	2 ± 0.2	90 ± 8	222 ± 31	0.1 ± 0.1
SHP-77	5 ± 1	2 ± 1	12 ± 2	0.1 ± 0.1
BHE	1 ± 0.1	1 ± 0.2	8 ± 2	0.1 ± 0.1
Lines with low *Src*-418 and high GJIC				
QUDB	7 ± 1	9 ± 2	20 ± 4	6.3 ± 1
SK-LuCi6	10 ± 1	8 ± 2	21 ± 4	6.5 ± 1
Rat F111	0.2 ± 0.1	0.2 ± 0.1	28 ± 3	5.1 ± 1
T51B	5 ± 1	5 ± 3	18 ± 2	6.0 ± 1
E10	6 ± 1	6 ± 4	26 ± 9	6.0 ± 1

^α^ Stat3-705 and *Src*418 levels were measured by Western blotting. Numbers represent relative values obtained by quantitation analysis, with the average of the values for *Src*-transduced, SK-LuCi6-*Src* cells taken as 100%. Averages of at least three experiments ± SEM are shown. For Stat3, data from cells grown to 50% confluence or 3 days after confluence are presented, with the average of the values for *Src*-transduced, SK-LuCi6-*Src* cells grown to 50% confluence taken as 100%. The transcriptional activity values obtained paralleled the Stat3-705 phosphorylation levels indicated [23]. Rat F111: Fisher Rat fibroblasts [48]; T51B: Rat liver epithelial line [36]; E10: mouse lung epithelial type II line [37]; ^β^ GJIC was assessed at 3 days after confluence.

### 4.3. Effect of Src and Stat3 upon GJIC in NSCLC Lines

**Figure 2:**
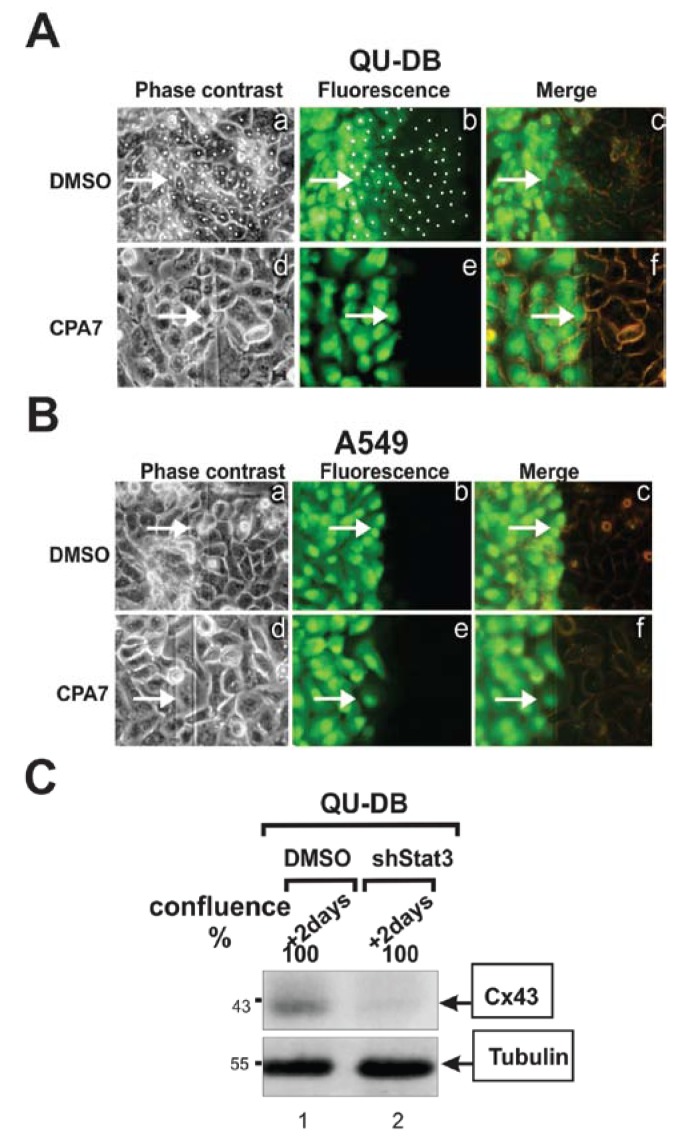
(**A**) Stat3 downregulation eliminates gap junctional permeability in human lung carcinoma QU-DB cells. QU-DB cells were plated in electroporation chambers and subjected to a pulse in the presence of Lucifer yellow, following treatment with the DMSO carrier alone (**a**–**c**), or CPA7 (**d**–**f**). After washing away the unincorporated dye, cells from the same field were photographed under fluorescence (**b**, **e**) or phase contrast (**a**, **d**) illumination. In (**b**), cells at the edge of the conductive area, which were loaded with LY through electroporation were marked with a star, and cells at the non-electroporated area which received LY through gap junctions were marked with a dot. Arrows point to the edge of the electroporated area. (**c**, **f**): Overlay of phase-contrast and fluorescence. Magnification: 240×. Note the extensive gap junctional communication in **b**; (**B**) Stat3 downregulation does not increase gap junctional permeability in human lung carcinoma A549 cells. Same as above, A549 cells. Note the absence of GJIC, even after Stat3 downregulation (**e**). From reference [33], reproduced with permission. Bar, 50 μm; (**C**) Stat3 downregulation reduces Cx43 levels. QU-DB cells, infected with a lentiviral vector carrying a Stat3-specific shRNA (lane 2) or not infected (lane 1), were grown to 2 days post-confluence and lysates probed for Cx43 or tubulin as a loading control (adapted with permission from references [33] and [38]).

An inverse relationship between *Src* and GJIC was noted in certain NSCLC lines [33]; A549, SK-Lu1, Calu-1, Calu-6, SW-900, SW-1573, WT-E, BEN, H1299, FR-E or SK-MES cells with high *Src*418 had low or undetectable gap junctional permeability, while two lines with very low *Src* levels (QU-DB, SK-LuCi6) had extensive GJIC (Figure 2 and Table 1). In addition, primary cells explanted and cultured from three lung cancer specimens had elevated *Src*418 and low GJIC [33]. These data are in line with the established role of *Src* as a GJIC suppressor. However, lines LC-T, BH-E and SHP-77 with low *Src*418 were found to have very low GJIC as well (Table 1), indicating that other, *Src*-independent factors may play a role in reducing gap junctional permeability in these cells. 

Examination of the role of Stat3 through the use of the CPA7 [40] or S3I-201 [49] inhibitors in the NSCLC lines with high *Src*418 showed that this treatment did not increase GJIC (e.g., line A549, Figure 2B) [33,38], in agreement with observations from T51B-*Src* cells [36], indicating that Stat3 is not part of a *Src* pathway to GJIC suppression. In addition, Stat3 inhibition with CPA7 or S3I-201 in LC-T cells which display high Stat3-705 despite the fact that *Src*418 levels are low, did not increase GJIC, indicating that Stat3, which is likely activated by a *Src*-independent mechanism, is not part of a pathway leading to gap junction closure in these cells. Finally, Stat3 inhibition in BH-E and SHP-77 cells that have very low GJIC although both *Src* and Stat3 phosphorylation levels are low did not reinstate junctional permeability, pointing to a mechanism of gap junction closure which may be independent from the *Src*/Stat3 axis altogether [38]. 

Examination of the effect of Stat3 inhibition upon GJIC levels in QUDB and SK-LuCi6 cells which have extensive communication indicated that Stat3 downregulation essentially abolished GJIC (Figure 2A) and Cx43 (Figure 2C), in agreement with results from T51B cells. Therefore, rather than increasing GJIC, Stat3 inhibition eliminates gap junctional permeability, that is Stat3 activity is actually required for gap junction function in established rodent lines, as well as in certain NSCLC lines which display extensive GJIC.

## 5. Discussion

Extensive data from a number of labs demonstrated that oncogenes such as mT, *Src* or Ras can suppress GJIC [12,50]. Moreover, the results revealed that lower levels of these proteins were sufficient to eliminate gap junction function than the levels necessary for full transformation [9,51], indicating that a decrease in GJIC may be an early event in neoplastic conversion. Therefore, the question arises on the role of *Src*, an oncogene often activated in cancer, and its effector Stat3 upon GJIC. 

**Figure 3:**
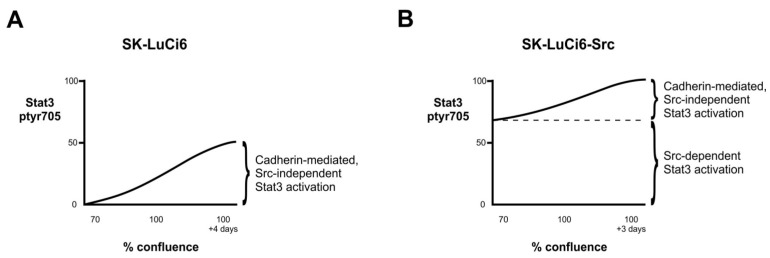
Schematic of Stat3 activity levels as a function of density in SK-LuCi6 (**A**) *vs.* SK-LuCi6-*Src* (**B**) cells. In SK-LuCi6 cells the cadherin-triggered, Stat3 activation is resistant to *Src* inhibition. In transformed SK-LuCi6-*Src* cells however, there are two pathways of Stat3 activation: v*Src*-dependent, same at all densities, and the cadherin-mediated, which is independent of *Src* and increases dramatically with confluence. The cadherin-mediated increase is not as pronounced however, due to the adverse effect of ***Src*** upon cadherins [50].

Examination of the relationship between *Src* and Stat3 must take cell density into account, since confluence of cultured cells itself causes a dramatic increase in Stat3, ptyr-705 phosphorylation and transcriptional activity (reviewed in [25,26]). However, despite the fact that *Src* is a potent Stat3 activator, *Src* is not involved in the density-dependent Stat3 upregulation. Therefore, at any given time-point Stat3-705 levels are the sum of the activation due to density alone (E10, T51B, QU-DB, SK-LuCi6), or density plus the Stat3 activation triggered by *Src* and/or other kinases (E10-*Src*, T51B-*Src*, SK-LuCi6-*Src*, LC-T, Calu-1, Calu-6, SW-900, SK-Lu1, A549, FR-E, WT-E) (Figure 3). Interestingly, activation of Stat3 through cadherin engagement was found to occur despite the presence of *Src*, which is known to induce cadherin degradation [52]; apparently, the residual cadherin present is able to activate Stat3 [53].

### 5.1. Stat3 Does Not Transmit Src Signals to Gap Junction Closure

Several signal transducers besides Stat3 are known to be downstream effectors of the *Src* kinase such as Ras/Raf/Erk, PI3k/Akt, the Crk-associated substrate (Cas) and others [54]. Constitutively active Ras is neoplastically transforming and can suppress GJIC [12,51]. Examination of the mechanism of *Src*-mediated GJIC suppression indicated that inhibition of Ras in *Src*-transformed, rat fibroblasts reinstated gap junctional communication [17]. Conversely, mT expression in Ras-deficient cells did not suppress GJIC [55]. Taken together, these data underline the importance of the Ras pathway in GJIC reduction by activated *Src*. It was also shown later that Cas is required for the *Src*-induced, reduction in gap junctional communication [56]. In sharp contrast, Stat3 inhibition did not restore GJIC in any of the lines examined, indicating that a role of Stat3 in the *Src*-induced, GJIC suppression in these cells is unlikely, despite the fact that constitutively active Stat3 can act as an oncogene and transform established lines [19].

### 5.2. Stat3 Plays a Positive Role in Gap Junctional Communication

The fact that cell density upregulates Stat3 concomitant with an increase in both Cx43 and GJIC prompted us to explore a potential positive role of Stat3 upon GJIC. Interestingly, Stat3 inhibition in non-neoplastic rodent fibroblasts and epithelial cells, as well as two NSCLC lines which exhibit extensive junctional communication (QU-DB, SK-LuCi6) abolished GJIC, indicating that Stat3 is required for the maintenance of gap junction function. 

Examination of the mechanism of GJIC suppression in NSCLC revealed an inverse relationship between *Src*, tyr-418 phosphorylation levels and GJIC in a number of lines. Since *Src* is known to suppress gap junctional communication it is tempting to speculate that *Src* may be responsible, at least in part, for gap junction closure in these lines. Therefore, since Stat3 is a positive regulator of GJIC and Cx43 levels, it appears that *Src* has a dual role upon GJIC; as an inhibitor, through Cx43 phosphorylation, but also as a GJIC activator, through Stat3 stimulation. However, the former prevails, with gap junction closure as a result. 

Stat3 downregulation caused apoptosis in all lines examined, which was more pronounced at 3 days post-confluence [33,57], the time of GJIC analysis. This hints at a link between GJIC reduction and apoptosis induced by Stat3 inhibition. In fact, results from a number of labs demonstrated that Stat3-705 activates a number of anti-apoptotic genes, such as BcL-xL, Mcl-1, survivin and Akt1 [21]. In addition, Stat3 can also inhibit apoptosis by downregulating mRNA’s of mitochondrial genes, thereby reducing oxidative phosphorylation and ROS (reactive oxygen species) production [58], while ser727-phosphorylated Stat3 enhances the activity of ETC (electrotransfer chain) complexes and glycolysis, and opposes the mitochondrial permeability transition pore, thereby inhibiting apoptosis further [59,60,61]. Global induction of apoptosis with etoposide, cycloheximide or puromycin was shown to lead to a loss of cell coupling, probably due to caspase-3-mediated degradation of Cx43, in primary bovine lens epithelial and mouse NIH3T3 fibroblasts [62]. In fact, Stat3 inhibition in cells transformed by *Src* or the Large Tumor antigen of Simian Virus 40 led to apoptosis [24,33,63], possibly due to activation of the transcription factor E2F family, potent apoptosis inducers, by these oncogenes. Therefore, apoptosis induced by Stat3 downregulation in cells with high *Src*/E2F activity may have accentuated gap junction closure. In lines such as LC-T which has low *Src*418, other oncogenes that activate Stat3, may also be activating the E2F family and induce apoptosis, with gap junction closure as a result. The fact that another major survival pathway, the PI3k/Akt also has a positive effect upon GJIC [64] tends to favor this interpretation. However, since Stat3 binds to and activates the Cx43 promoter, it is possible that Stat3 inhibition may downregulate Cx43 through a direct effect upon its mRNA as well [65,66,67].

In non-transformed cells with low E2F levels, grown to low densities, Stat3 inhibition was previously shown to cause merely a growth retardation. However, at high densities, such as needed for optimal gap junction formation, Stat3 inhibition leads to apoptosis [57]. This could be due to the high levels of LATS (large tumor suppressor) kinase activity which is induced by the mechanical constraints at high confluence. LATS was shown to downregulate the transcription factors YAP/TAZ (Yes associated protein/transcriptional coactivator with PDE binding motif), which are potent survival signals [26]. Therefore, apoptosis induction through a reduction in Stat3 levels or activity could explain the dramatic reduction in Cx43 and GJIC upon Stat3 pharmacological or genetic inhibition, in lines with low *Src*/E2F. 

## 6. Conclusions

Although Stat3 is generally growth promoting, and in an activated form it can act as an oncogene, it does not transmit gap junction suppressing signals in any of the NSCLC cell lines examined. This holds true for cells where *Src* may have been responsible at least in part for GJIC suppression like the majority of NSCLC lines, but also for lines like LC-T where *Src* levels were found to be low, that is other oncogenes must be responsible for gap junction closure. In the contrary, Stat3 is required for gap junctional communication and the maintenance of Cx43 levels, both in normal epithelial cells and in certain tumor lines that retain GJIC. In cells such as BH-E the gap junction closure may be independent from the *Src*/Stat3 axis altogether.

The role of connexins in cancer is complex. Numerous models support the idea that connexins are tumor suppressors at the early stages of carcinogenesis. However, recent evidence indicated a positive role for connexins in facilitating metastasis ([68,69,70], reviewed in [71]). Therefore, inhibition of the Stat3/Cx axis might be beneficial at late stages of cancer progression. This novel role of Stat3 in gap junction function may be an important regulatory step in the progression of tumors that exploit such a pathway. 
